# Epigenetic interplay between methylation and miRNA in bladder cancer: focus on isoform expression

**DOI:** 10.1186/s12864-021-08052-9

**Published:** 2021-10-21

**Authors:** Manu Shivakumar, Seonggyun Han, Younghee Lee, Dokyoon Kim

**Affiliations:** 1 Department of Biostatistics, Epidemiology and Informatics, Perelman School of Medicine, University of Pennsylvania, Philadelphia, PA USA; 2 Institute for Biomedical Informatics, University of Pennsylvania, Philadelphia, PA USA; 3 Department of Biomedical Informatics, University of Utah School of Medicine, Salt Lake City, USA; 4 Huntsman Cancer Institute, Salt Lake City, USA

**Keywords:** Epigenetics, Bladder cancer, Methylation, miRNA, Isoform expression

## Abstract

**Background:**

Various epigenetic factors are responsible for the non-genetic regulation on gene expression. The epigenetically dysregulated oncogenes or tumor suppressors by miRNA and/or DNA methylation are often observed in cancer cells. Each of these epigenetic regulators has been studied well in cancer progressions; however, their mutual regulatory relationship in cancer still remains unclear. In this study, we propose an integrative framework to systematically investigate epigenetic interactions between miRNA and methylation at the alternatively spliced mRNA level in bladder cancer. Each of these epigenetic regulators has been studied well in cancer progressions; however, their mutual regulatory relationship in cancer still remains unclear.

**Results:**

The integrative analyses yielded 136 significant combinations (methylation, miRNA and isoform). Further, overall survival analysis on the 136 combinations based on methylation and miRNA, high and low expression groups resulted in 13 combinations associated with survival. Additionally, different interaction patterns were examined.

**Conclusions:**

Our study provides a higher resolution of molecular insight into the crosstalk between two epigenetic factors, DNA methylation and miRNA. Given the importance of epigenetic interactions and alternative splicing in cancer, it is timely to identify and understand the underlying mechanisms based on epigenetic markers and their interactions in cancer, leading to alternative splicing with primary functional impact.

**Supplementary Information:**

The online version contains supplementary material available at 10.1186/s12864-021-08052-9.

## Background

Cancer is a complex disease that is caused by alterations in the genome and epigenome. The alterations in cancer are different in each person as the tumor accumulates additional changes occur. As a result, the genetic and epigenetic changes in the same tumor could be different among diverse cells. Precision medicine is an emerging approach to the treatment of cancer by developing targeted therapies taking into account patients’ environmental, lifestyle and genomic variabilities [1] . To apply a precision medicine approach to cancer, the fundamental understanding of genomic and epigenetic abnormalities that cause carcinogenesis and drive its progression is essential. Understanding the epigenetic abnormalities is very challenging, as various epigenetic machinery interacts with each other in an integrated manner to maintain global expression pattern [2]. Thus, many large-scale collaborative initiatives have been undertaken to generate large multi-omics datasets in cancer like The Cancer Genome Atlas (TCGA) and The International Cancer Genome Consortium (ICGC), and multiple data integration methods have been developed to understand multi-omics markers associated with clinical outcomes [3–13].

Many abnormalities in cancer are caused by epigenetic changes in DNA methylation and microRNA (miRNA) [14, 15]. Previously, we identified interactions with methylation and miRNA that were associated with gene expression and survival outcome [13]. However, methylation and miRNAs are also known to play a role in isoform usage in cancer [16]. In another study, we looked at the effects of alternative splicing (AS) on miRNA binding sites in bladder cancer to conclude that understanding transcript isoforms is essential to understand gene regulatory mechanisms mediated by miRNA [17].

Alternative splicing is an underlying contributor to biological complex and differences. Each gene in eukaryote cell is composed of two distinct blocks of sequences, exons and intron. As exon is the region encoding segments of the protein, exon is included, and introns are removed by alternative splicing mechanism during transcription. Some exons are also selective, which means that some exons may also be removed from the nascent mRNA, leading to a different combination of exons in the final transcript and are also implicated in a variety of human diseases [18–20]. ~ 95% of human genes are alternatively spliced. Conservative estimates of AS show that at least 50% of exons are alternatively spliced [21]. That is, most genes can each produce an entire array of potentially unique proteins. Even if the same genes are actively transcribed in two different cells, their proteins can be different depending on how those genes are spliced.

As the regulatory molecules (i.e., miRNA and methylation) are a type of epigenetic factors affecting gene expression [22], gene regulation and biological complexity may be more complicated when alternative splicing interact with these regulatory molecules (i.e., methylation and miRNA). Cancer genes are down- or up-regulated by the methylation status in promoter regions, hyper- methylations in promoter regions of oncogenic genes and hypo-methylated in promoter regions of tumor suppressor genes, respectively [23]. Due to the nature of GC contents; higher in exon compared to intron and higher in constitutive exon compared to alternative splicing exon, methylation may be differentiated across exons and introns by splicing status [24–26]. Furthermore, hypo-methylated intron has been shown to be more retained in breast cancer patients [27]. In the purpose of integrating methylation with genetic regulation, EpiMethEx [28] is one of the well-developed tools that directly associate methylation with transcript isoform.

In addition to methylation, splicing occurring in 3′ UTR may affect regulatory effect of miRNA [29]. When exon encompassing miRNA binding site is skipped (i.e., exon skipping event) or partial exon is alternatively spliced (i.e., 5′ or 3′ splice site event), given mRNA maybe not be repressed by miRNA [17]. Reversely, inclusion of new exon or intron (i.e., retained intron event) may potentially provide additional miRNA binding site resulting in a reduced amount of mRNA product [17].

**Fig. 1:**
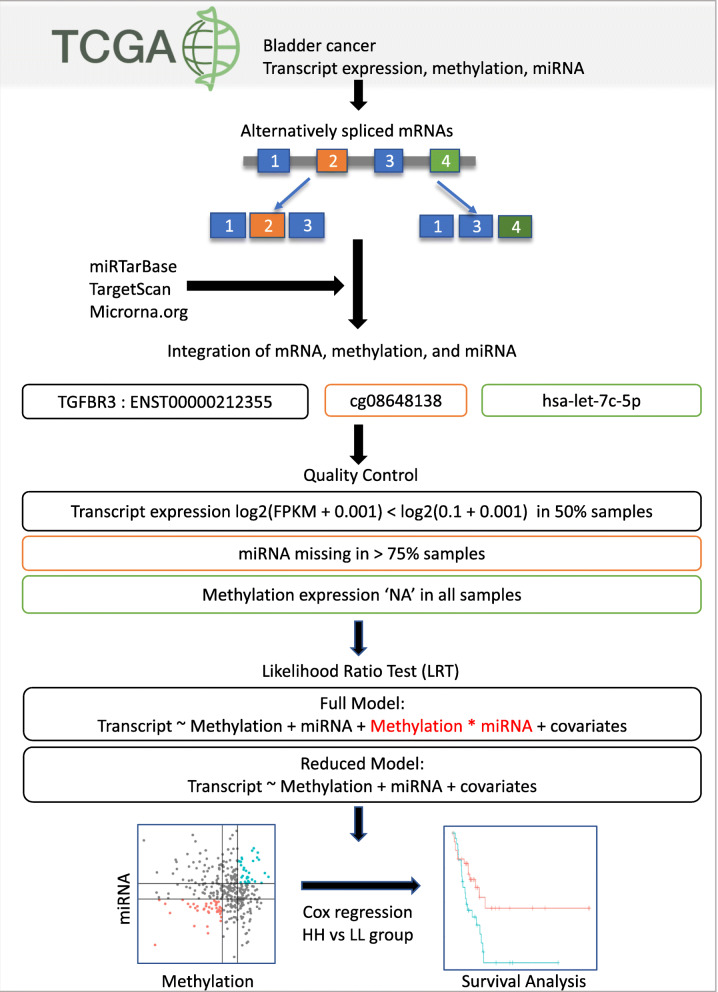
Overview of the study. The diagram illustrates various steps in the study - formation of isoform, miRNA and methylation pairs, data quality control, LRT and survival analysis

In our study, we seek to study epigenetic interactions (i.e., miRNA and methylation) in gene regulation through alternative splicing. We classified the interaction into two terms, synergistic and antagonistic in conjunction with alternative splicing status. We then evaluated whether the differences in isoform expression resulting from epigenetic interactions are associated with survival (Fig. 1). Thus, in this study, we put forward a method to identify the effects of methylation and miRNA interaction associated with isoform expression and its further association to survival.

## Results and discussion

### Methylation and miRNA interaction associated with isoform expression

After applying the likelihood ratio (LRT) test on the full and reduced model, 136 out of 2,561,305 combinations were found to be significant (Bonferroni adjusted *p*-value < 0.05). Altogether, there were 61 unique isoforms, 105 methylation probes and 51 miRNAs across 56 genes. The number of samples varied across each combination, with a minimum of 294 and mean of 388.3, due to missing values. All the significant combinations, number of samples, beta values from the full model, correlation between isoform, methylation, miRNA pairs, and Cox regression *p*-values are provided in Table S1. The distribution of the direction of effect determined by beta values is shown in Table 1. It can be noted from Table 1 that 59 methylation probes have a negative direction of effect, and 77 methylation probes have a positive direction of effect, holding 43%. However, in the case of miRNA about 76% (103/136) of miRNAs have the negative direction of effect, indicating most of them downregulate isoform expression. Additionally, Amuran et al. compiled a list of 106 miRNAs associated with bladder cancer by reviewing the literature and about 71% (97/136) miRNAs in the combinations were present in the list of miRNAs deregulated in bladder cancer [30].

**Table 1:** Distribution of direction of effect of methylation, miRNA and interaction term. (+) for synergistic and (−) for antagonistic effect

Methylation	miRNA	Interaction	# combinations	# Cox-regression
–	–	–	10	2
–	–	+	34	2
–	+	–	11	1
–	+	+	4	1
+	–	–	8	0
+	–	+	51	4
+	+	–	16	3
+	+	+	2	0

### Patterns of methylation and miRNA interaction associated with isoform expression

**Fig. 2:**
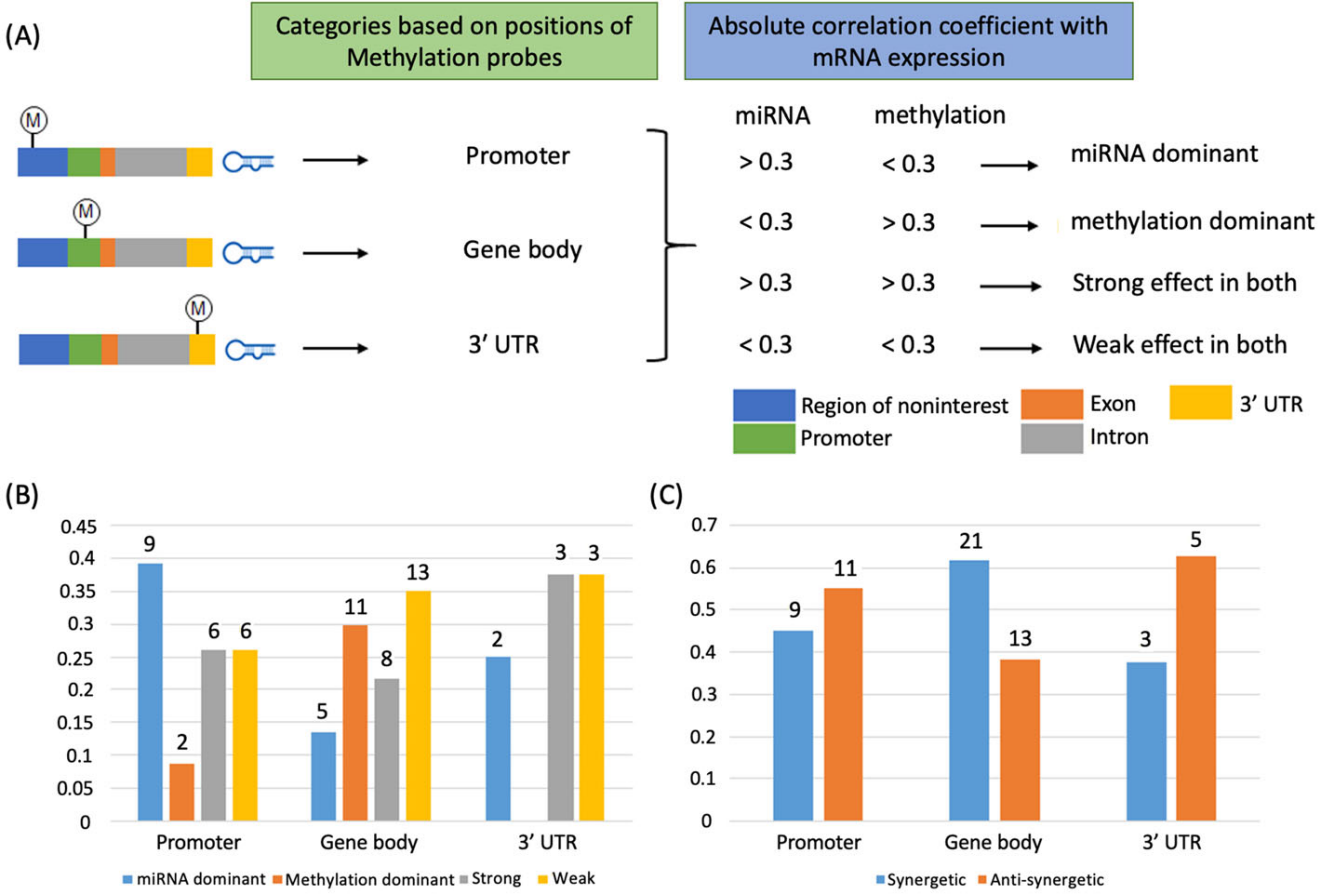
Categorization of interactions based on methylation probes and correlation coefficient. **A** Our criteria for categorizing interactions. **B** Distribution of interactions according to the methylation probe position. **C** Distribution of synergistic or antagonistic interaction

We stratified the interaction pattern according to methylation probe’s location; promoter, gene body, and 3’UTR. 44 methylations (19 unique transcripts), 66 methylations (32 unique transcripts) and 10 methylations (8 unique transcripts) were located in the promoter, gene body and 3’UTR regions, respectively. In general, 3′ UTR is considered part of gene body, but we separated 3′ UTR region from gene body as miRNA can directly interact with methylation in 3′ UTR region only, as miRNA binds to 3’UTR region. The interaction is first defined as a methylation-dominant or miRNA-dominant (See Methods). As shown in Fig. 2B, interactions in promoter region (i.e., a location of methylation probe) was more likely to have miRNA-dominant regulation. Notably, very few interactions had methylation -dominant cases (less than 10%). However, interestingly methylation exerted dominant regulation effect in gene body. We then further stratified the interactions, synergetic- or antagonistic- effect. Interestingly, the antagonistic effect was observed little more than synergetic in the promoter and 3′ UTR region. On the other hand, a higher number of synergetic effect combinations were observed in the gene body (Fig. 2C).

### Differential isoform expression and association with survival outcome

We were interested in understanding how the low/high expression of methylation and miRNA expression in pairs significantly associated with isoform expression altered the isoform expression. So, we split samples were split into LL and HH groups. To further understand the implication of these changes on survival of the patients we performed Kaplan Meier survival analysis between the groups. The differential isoform expression between HH and LL (2-group test) was significant for 100 combinations (out of 136) at (t-test *P* < 0.05). Further, the differential expression between 4 groups HH, LL, HL, and LH (4-group test) were also examined, and 98 combinations were significant at (ANOVA *P* < 0.05). In addition to that, 126 combinations were significant in either 2-group test or 4-group test, and 76 combinations were significant in both 2-group test and 4-group test. The results show that for most of the combinations that have significant interaction between methylation and miRNA, the differential expression can be observed between HH, LL, HL, and LH groups. Subsequently, the samples were split into HH and LL groups to determine if there is any difference in survival of patients between the groups. Cox regression was run on 136 significant combinations. Out of 136 combinations, 13 were associated with survival outcome at Cox *p*-value threshold < 0.05 (Table 2). Of the 13 combinations that were significantly associated with survival outcome, isoforms from 11 combinations were differentially expressed between HH and LL groups and the other two isoforms between the LL, HH, LH and HL groups. The isoform expression was significantly higher in LL group in 7 of the 13 combinations and lower expression in the four remaining combinations (Fig. S1-S13).

**Table 2:** Combinations associated with survival between HH and LL group

Isoform	Methylation	miRNA	LRT *p*-value	Cox p-value^a^	Higher expression^b^	Higher survival rate^c^
SGCD_ENST00000435422	cg19748027	hsa-miR-409-3p	5.24E-10	0.0013	HH	LL
PLS1_ENST00000457734	cg05652551	hsa-miR-142-5p	9.10E-10	0.0014	LL	LL
CAV1_ENST00000341049	cg04474049	hsa-miR-194-5p	1.45E-08	0.0049	LL	HH
PLS1_ENST00000457734	cg05652551	hsa-miR-155-5p	7.07E-12	0.0060	LL	LL
HID1_ENST00000425042	cg07430967	hsa-miR-125a-5p	1.50E-12	0.0072	HH	HH
TGFBR3_ENST00000212355	cg08648138	hsa-let-7c-5p	9.24E-13	0.0076	HH	LL
PMEPA1_ENST00000341744	cg01515444	hsa-miR-200a-5p	2.20E-10	0.0117	LL	HH
H2AFY_ENST00000304332	cg01874869	hsa-miR-100-5p	1.24E-08	0.0139	–	HH
THBS2_ENST00000366787	cg19681793	hsa-miR-105-5p	1.29E-09	0.0186	LL	HH
RND3_ENST00000263895	cg17730764	hsa-miR-200c-3p	9.19E-09	0.0196	–	LL
ACOT7_ENST00000377855	cg16429975	hsa-miR-155-5p	1.82E-08	0.0271	HH	HH
TMTC3_ENST00000266712	cg07537152	hsa-miR-98-5p	6.13E-09	0.0342	LL	HH
SCD5_ENST00000319540	cg09031823	hsa-miR-200a-3p	2.56E-11	0.0385	LL	HH

^a^The p-value from cox regression between LL and HH groups

^b^The group which has significantly higher isoform expression (t-test *P* < 0.05)

^c^The group which has higher survival rate (Cox regression *P* < 0.05)

## Case study: CAV1, TGFBR3, and RND3

**Fig. 3:**
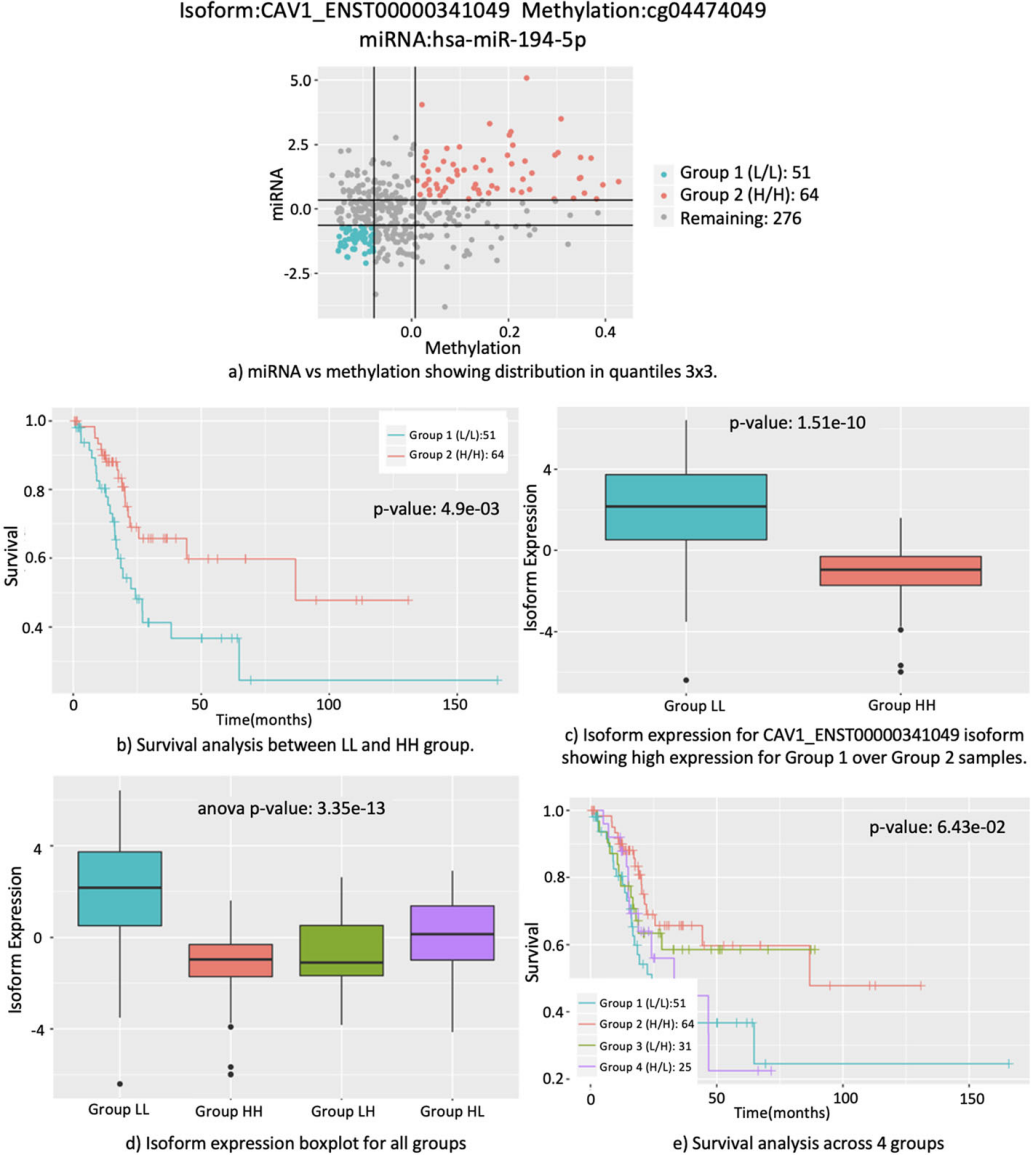
Plots for combination of CAV1_ENST00000341049, cg04474049 and hsa-miR-194-5p. **a**) The samples were divided into 9 groups based on 3 quintiles of methylation and miRNA. **b**) Kaplan-Meier survival curve between groups LL and HH. The survival among the groups was significantly different with Cox regression *p*-value 1.51 × 10^−10^. **c**) Boxplot showing isoform expression of group LL and HH, which are significantly different (t-test p-value 1.78 × 10^− 11^). **d**) Boxplot of isoform expression between groups LL, HH, LH, HL. **e**) Kaplan-Meier survival curve for groups LL, HH, LH and HL. The Kaplan-Meier log rank p-value for the combination was 6.43 × 10^−02^

Figure 3 shows plots for isoform ENST00000341049 in gene *CAV1*. *CAV1* is known to be associated with high-grade bladder cancer as an oncogenic membrane protein, and its overexpression is known to be associated with bladder cancer progression [31, 32]. As observed in Fig. 3c, the isoform has significantly higher expression in the LL group (Fig. 3a, red points) than in the HH group (Fig. 3a, cyan points). Since higher expression of *CAV1* is associated with cancer progression, the survival rate should be lower for LL group. As anticipated, the survival rate was significantly lower (Cox *p*-value < 4.9 × 10^− 3^) for LL group (Fig. 3b). That is, disruption in regulation of methylation (i.e., cg04474049) and miRNA (i.e., hsa-let-7c-5p) may contribute to bladder cancer progression.

**Fig. 4:**
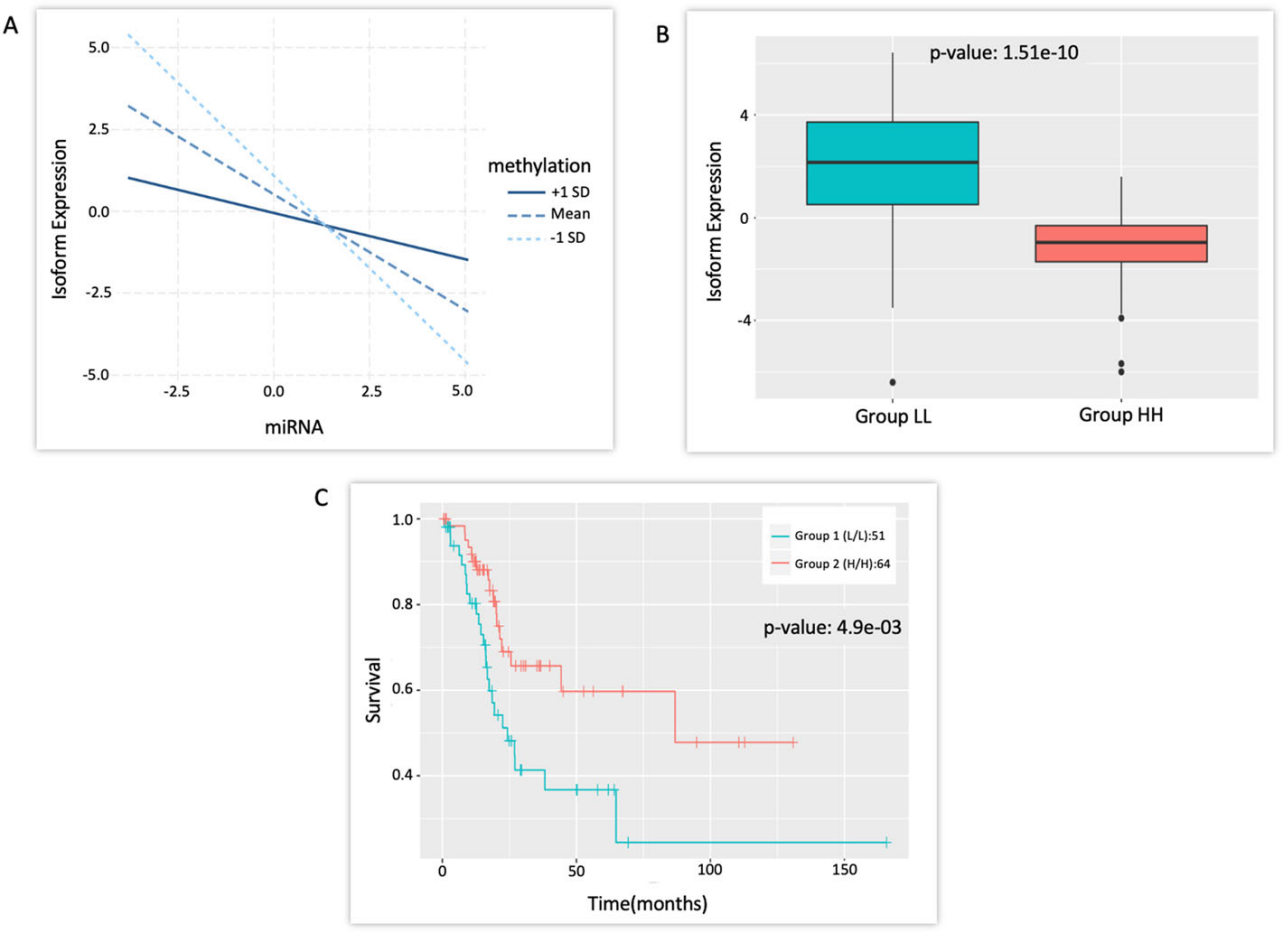
ENST00000212355 (gene TGFBR3) isoform, hsa-let-7c-5p miRNA and cg08648138 methylation probe interaction and survival outcome

One of the isoforms, ENST00000212355 (gene *TGFBR3*) from a combination associated with survival, is targeted by miRNA - hsa-let-7c-5p, which is known to be a tumor suppressor and acts by downregulating *TGFBR3* post transcriptionally [33] (Fig. 4a). Moreover, *TGFBR3* knockout is known to reduce tumor size. From the interaction plot in Fig. 4b, it can be observed that the isoform expression is low when methylation and miRNA are both low. However, when methylation is higher (+ 1 sd), the isoform expression decreases with an increase in miRNA expression, but the slope is comparatively smaller. Thus, the LL group has lower isoform expression than the HH group as seen in Fig. 4c. Consequently, LL group has a higher survival rate as compared to the HH group (Fig. 4d). The other isoforms ENST00000425042 and ENST00000263895 of *HID1* and *RND3* respectively are part of combinations that are associated with survival. *HID1* and *RND3* are known to be downregulated in various cancers [34, 35]. The loss of function of *HID1* is known to be associated with the development of cancer [34]. Consistent with the literature, it was observed that the LL group has a significantly lower expression of isoform ENST00000425042 and also a lower rate of survival (Fig. S5).

*RND3* is known to be downregulated by its target miRNA, hsa-miR-200c-3p, in the combination. The downregulation of *RND3* leads to higher expression of *CCND1*, which can lead to oncogenesis and tumor progression [35]. Besides, some of the genes could also show oncogenic properties in cancer. Two other genes that are part of combinations associated with survival, *PMEPA1* and *THBS2* are known to be upregulated in cancer [36, 37]. Moreover, *PMEPA1* knockout is known to impair tumor growth, and *THBS2* overexpression is known to be associated with vascular invasion, advanced primary tumor status and nodal metastasis [37]. Fatty acids play an important role in cancer cells, as cancer cells need large amounts of fatty acids to grow [38]. Thus, fatty acid metabolism is involved in cancer progression. Two of the genes, *ACOT7* and *SCD5,* with isoforms ENST00000377855 and ENST00000319540 respectively, are part of the “Biosynthesis of unsaturated fatty acids” pathway (KEGG 2019 Human). The pathway was also significantly enriched (*p*-value = 0.00011 and adjusted p-value = 0.035) based on the enrichment test run using genes of all isoforms from combinations that were associated with survival, using Enrichr [39, 40].

### Methylation and miRNA interaction patterns

Many different interaction patterns of miRNA and methylation associated with isoform expression were observed. Especially, more miRNA dominant interactions were observed in promoter region, and more methylation dominant interactions were observed in gene body region. In fact, methylation within promoter region alters gene expression by affecting binding of transcription factors, and its regulation prior to that of miRNA. In other words, mRNA expression may be susceptible to be regulated by miRNA which is a next step of the methylation regulation. That is, the basis of this knowledge may contribute to more observation of dominant miRNA regulation with interactions with methylation within promoter region. Unexpectedly, in methylation within gene body, we found more methylation dominant interaction. The Methylation within gene body is known to relate to splicing processing, cause a temporal pause of the transcription process that help correct splicing [25]. Splicing regulation is very complex and occur generally at the mRNA processing after gene expression regulation [41]. Thus, these methylations may affect mRNA expression regulation more constantly than methylation in promoter. Although we separated methylation in 3′ UTR from gene body to understand patterns when interaction of miRNA and methylation occurred in the same location, we did not find distinct characteristics. However, it may be caused by a small number of interactions in the case. In addition, there was also different patterns of synergistic and antagonistic effect between methylation in promoter and gene body (Fig. 2B).

As we discussed above, we observed the uneven distribution of the interactions across gene regions (Fig. 2B). To verify if this difference may be due to the unique enrichment of underlying distribution of methylation in certain gene regions for interactions with miRNA or not, we counted the number of underlying methylation probes in each gene region; promoter, gene body, and 3′ UTR. The methylation was most counted in gene body region (325,147 probes), which is followed by promoter (205,175 probes) and 3′ UTR region (26,228 probes) (Fig. S14), in which the underlying distribution can be biased by the length of each region: gene body is the longest in length. Taken together, we found that the 3′ UTR has the smallest number of the underlying methylation but the most enrichment of the interaction with miRNAs, suggesting that methylations interacting with miRNA may be enriched in 3′ UTR which they are co-localized.

**Fig. 5:**
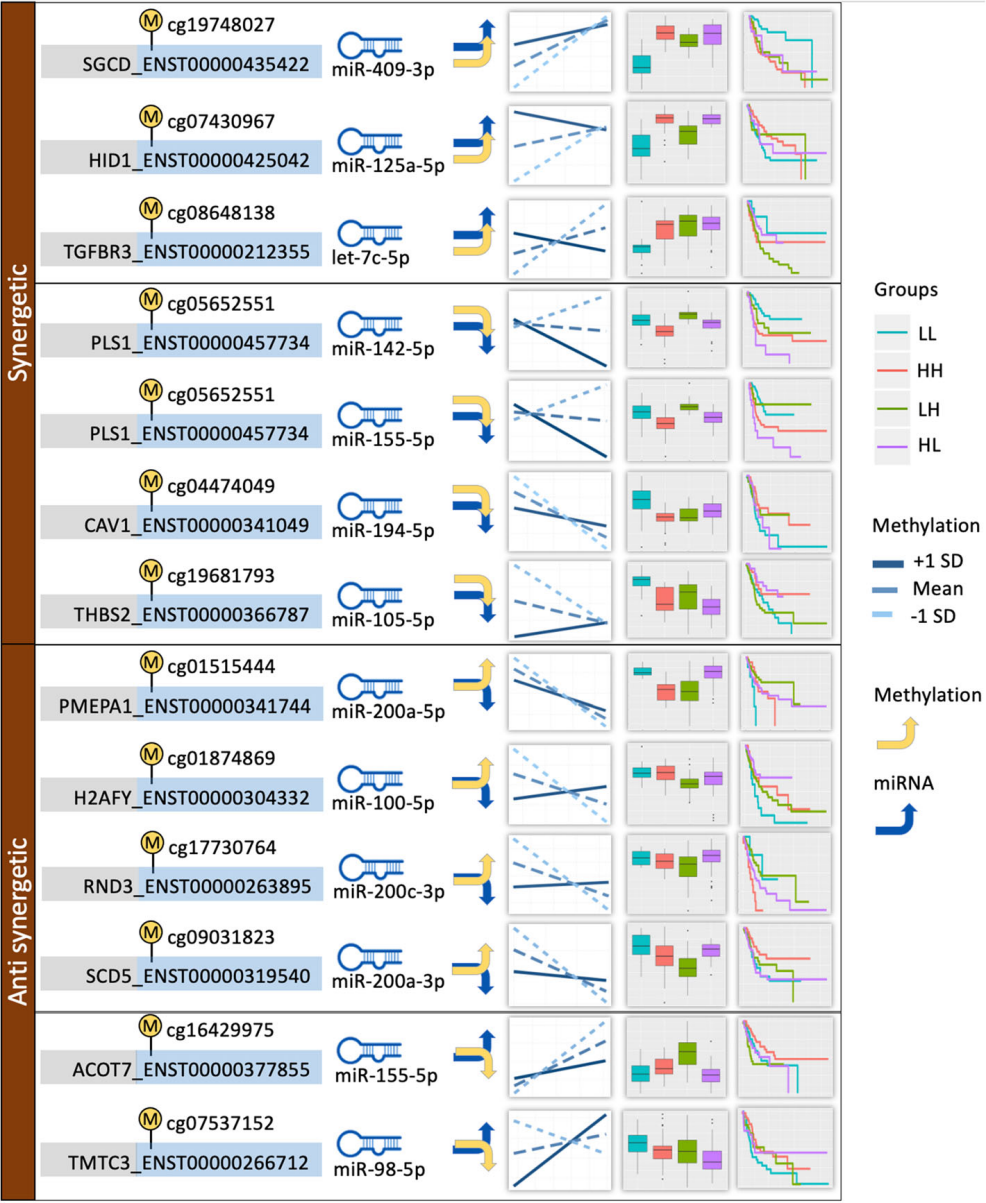
Synergetic and antagonistic interactions. The blue arrow shows the correlation between miRNA and isoform and yellow arrow shows correlation between methylation and isoform. The x-axis for the line plot represents miRNA expression and y axis represents isoform expression. The lighter of the 3 lines is methylation − 1 SD, the dark solid line is +1SD and the medium one is the Mean methylation. The boxplot shows plot of groups LL, HH, LH and HL from left to right

Out of 13 significant combinations associated with survival, seven combinations were synergetic interaction and remaining six combinations were antagonistic interactions. Particularly in synergetic combinations, three combinations had positive methylation and miRNA correlation, and the remaining four combinations had negative methylation and miRNA correlation. Figure 5 summarizes all the combinations into synergetic and antagonistic categories. Additionally, if the four groups were divided based on synergetic and antagonistic effect, it can be observed in Fig. 5 that most of the isoform expression between groups LL, HH, LH, and HL are similar in the same group. For instance, SGCD_ENST00000435422, HID1_ENST00000425042 and TGFBR3_ENST00000212355 have synergetic effect with methylation and miRNA being positive. The isoform expression between LL, HH, LH, and HL groups are similar. Specifically, all three had lower isoform expression in LL group and higher isoform expression in other groups. The opposite also holds true in case of 2nd group with negative synergetic interaction where isoform expression is higher in LL group for all four combinations as compared to other groups.

As we showed that a combination of methylation and miRNA could provide an improved knowledge of the genetic regulation underlying bladder cancer and the methylation in this study and miRNA pattern is a unique characteristic across cancer types [42, 43], our approaches could be expanded to other cancers if the matched data (methylation and miRNA) is available.

## Conclusion

In this study, we considered the use of TCGA bladder cancer data to show epigenetic interactions between methylation and miRNA associated with survival in bladder cancer. The method used successfully identified 136 significant methylation and miRNA interactions that were associated with isoform expression. Further, out of 136 significant interactions, 13 were significantly associated with survival. Further, it also observed that a greater number of miRNA dominant interactions were observed in the promoter region whereas, a greater number of methylation dominant interactions were observed in the gene body. Additionally, isoform expression patterns were also observed with synergetic and antagonistic interactions. This shows that miRNA, methylation and isoform expression data can be used to study interactions between methylation and miRNA that are associated with survival outcome. Further, this study also shows different categorizations and patterns of interaction at various sites. The findings in this study could elucidate some of the complex epigenetic mechanisms involved in carcinogenesis and cancer progression, which could aid in the development of new targeted therapies and be valuable when applied to precision medicine.

## Methods

### Dataset and quality control

The Cancer Genome Atlas (TCGA) data was obtained from Xena browser (http://xena.ucsc.edu). Xena browser provides pre-compiled datasets derived from NCI’s Genomic Data Commons (GDC) public resource for further bioinformatics analysis [44]. The normalized isoform expression data using RSEM FPKM was downloaded from the TCGA Pan-Cancer (PANCAN) section of Xena browser. Further, the clinical data was also downloaded from the PANCAN section, as it contains the latest updated survival data. However, the methylation and miRNA data were obtained from TCGA Bladder Cancer (BLCA) section. The subset of BLCA patients was obtained for the isoform expression data using sample IDs from the clinical file. After excluding samples from “Solid tissue normal” as defined by TCGA sample type code “11”, there were 407 BLCA samples with isoform expression data, 415 with methylation data and 410 with miRNA data. Additionally, five samples were removed from clinical data because of missing, age at diagnosis, survival time, AJCC pathologic tumor stage, or histological grade, resulting in 409 samples. Consequently, the common samples between all four datasets were extracted, leading to a total sample count of 399. The clinical demographics for 399 samples are shown in Table 3. Further quality control (QC) steps were applied to each dataset. Initially, before QC, there were 198,619 isoforms, 485,577 methylation probes and 2588 miRNAs. Only isoforms that have FPKM threshold ≥0.1 in more than 50% of samples and genes with at least two transcript isoforms were selected for the analysis. Further, methylation probes with all ‘NA’ values were removed, and miRNAs with expression value missing in more than 75% of samples were removed. Finally, 67,627 isoforms, 396,065 methylation probes, and 706 miRNAs passed the QC criteria. Table 4 shows the data types, platform and number of features for each data type after QC. To better interpret the interactions, the isoform expression, methylation and miRNA expression values were centered by subtracting with respective mean. Additionally, any isoform, methylation and miRNA expression values that were not in the range [Q1–3 × IQR, Q3 + 3 × IQR] were considered outliers and were removed. Further information and links to the files used from Xena browser are listed in “Availability of data and materials” section.

**Table 3:** Clinical demographics of samples after QC

Sex	
Male	301
Female	108
Age at diagnosis	
Mean	68.04
Standard deviation	10.63
AJCC pathologic tumor stage	
Stage I	2
Stage II	130
Stage III	141
Stage IV	136
Histological grade	
High	388
Low	21
Vital Status	
Diseased	229
Alive	180
Average survival days	
Diseased	1021.43
Alive	553.78

**Table 4:** Data types, platform and number of features used

Data	Platform	# of features after QC
Isoform expression	Illumina HiSeq miRNA-Seq	67,627
Methylation	Infinium HM450 BeadChip	396,065
miRNA expression	Illumina HiSeq RNA-Seq	706

### Isoform, methylation and miRNA binding sites

Most of miRNA target predictions are dependent on sequence similarity between mature miRNA and 3’UTR of mRNA. These predictions have enormous false-positive rate due to lack of experimental validation. Thus, in order to obtain comprehensive and reliable miRNA target site information, reducing the false-positive rate, we combined miRNA target predictions from databases generated using two different prediction methods and one experimentally validated data set: 1) TargetScan (release 7.0) [45], 2) MicoRNA.org [46] which computationally predicted miRNA target sites based on conserved complementarity and the miRanda algorithm between targets of miRNAs and mRNAs, respectively and 3) miRTarBase that identified relations between miRNA and mRNA based on experimental validations through reporter assay, western blots, and etc. [47]. In particular, we only included predictions with high confidence scores (alignment score ≥ 120 and binding energy ≤ − 7.0) of miRanda algorithm from the MicoRNA.org database in this study. We carried out data quality control in three steps as follows: first, we tabulated miRNA and mRNA pairs using miRTarBase. Second, for these pairs, we obtained genomic coordinates of mRNA-target sites in 3′ UTR by matching miRNA IDs (i.e., hsa-miR-199) with TargetScan and MicroRNA.org databases. Third, we then annotated 3′ UTR regions of each mRNA with Ensembl reference (release version 75, (http://ftp.ensembl.org/pub/release-75/gtf/homo_sapiens/, September 2016). The total of 439,404 pairs of miRNAs and their target sites were obtained, and these pairs are 2.7% of the total predictions (16,228,619 pairs) reported in the MicroRNA.org. Further, the methylation probe ids from Xena were mapped to isoforms using gene names in the ID/Gene mapping file provided by Xena browser. Totally, there were 6,571,851 combinations which were further filtered to include only 67,627 transcripts from isoform dataset and 706 miRNAs from miRNA expression dataset. Finally, we ended up with 2,561,305 combinations that had isoform, methylation and miRNA expression data.

### Methylation and miRNA interaction

To identify the interactions between methylation and miRNA with respect to isoform expression, two linear models were used. The full model consisted of a linear combination of methylation, miRNA and an interaction term, whereas the reduced model only contained a linear combination of methylation and miRNA. However, both models were adjusted for the same covariates - age at diagnosis, gender, AJCC pathologic tumor stage, and histological grade. The covariates were obtained from the clinical dataset and, their distributions are shown in Table 3. Specifically, the full model was defined as: isoform-expression ~ methylation + miRNA + methylation * miRNA + covariates and the reduced model was defined as: isoform-expression ~ methylation + miRNA + covariates. The significance of the interaction was determined by applying LRT between the full and reduced model. Further, the LRT *p*-values were adjusted for multiple testing using Bonferroni correction, and any combination with Bonferroni corrected p-value < 0.05 was considered significant.

### Categorization of miRNA and methylation pairs based on methylation probe location

As shown in Fig. 2A, for the significant miRNA and methylation pairs, we divided them into three categories according to the location of methylation probes in intragenic regions, promoter (upstream 2000 bp), gene body (exons and introns), or 3’UTR. Then, for each category, we defined miRNA, methylation, or both dominant regulation of isoform expression based on the correlation coefficient value between either of miRNA or methylation and isoform expression. In other words, as shown in Fig. 2A, an absolute correlation coefficient value of miRNA greater than 0.3 and methylation less than 0.3 with isoform expression was considered as miRNA dominant regulation; on the other hand, the correlation coefficient value of miRNA less than 0.3 and methylation more than 0.3 with isoform expression was considered as methylation dominant. In addition, if an absolute correlation coefficient value of both miRNA and methylation have more than 0.3 or less than 0.3 with isoform expression, it was defined as strong effect and weak effect respectively.

### Determination of synergetic or antagonistic effect of methylation and miRNA interaction on mRNA expression

We defined as a synergistic or antagonistic effect of methylation and miRNA pairs on isoform expression based on correlation coefficient values. Synergistic effect on isoform expression is the case when the both has the same direction of correlation with isoform expression (i.e. positive/positive or reverse/reverse correlation), whereas antagonistic effect is the case when they have different directions (i.e., a positive/reverse).

### Survival analysis and differential isoform expression

To further investigate differential isoform expression and difference in survival of the patient groups with high/low methylation and miRNA expression, the samples were split into nine groups based on methylation and miRNA expression values together. The groups were created by splitting the methylation data into three quantiles and miRNA data into three quantiles, as shown in Fig. 2A. The two extreme groups – a group with high methylation and high miRNA expression (HH) and group with low methylation and low miRNA expression (LL) were selected to run overall survival analysis. The survival analysis was run using Cox regression, adjusting for covariates - age at diagnosis, gender, AJCC pathologic tumor stage, and histological grade. Any combination with cox regression *p*-value < 0.05 was considered significant. Further, the differential isoform expression was also analyzed between HH and LL group using the t-test. Additionally, differential isoform expression between HH, LL, LH, and HL groups were also analyzed using ANOVA. Any p-value from t.test and ANOVA below 0.05 was considered significant.

## Supplementary Information

**Additional file 1 Fig. S1.** Plots for combination SGCD_ENST00000435422, cg19748027 and hsa-miR-409-3p. **Fig. S2.** Plots for combination PLS1_ENST00000457734, cg05652551 and hsa-miR-142-5p. **Fig. S3.** Plots for combination CAV1_ENST00000341049, cg04474049 and hsa-miR-194-5p. **Fig. S4.** Plots for combination PLS1_ENST00000457734, cg05652551 and hsa-miR-155-5p. **Fig. S5.** Plots for combination HID1_ENST00000425042, cg07430967 and hsa-miR-125a-5p. **Fig. S6.** Plots for combination TGFBR3_ENST00000212355, cg08648138 and hsa-let-7c-5p. **Fig. S7.** Plots for combination PMEPA1_ENST00000341744, cg01515444 and hsa-miR-200a-5p. **Fig. S8.** Plots for combination H2AFY_ENST00000304332, cg01874869 and hsa-miR-100-5p. **Fig. S9.** Plots for combination THBS2_ENST00000366787, cg19681793 and hsa-miR-105-5p. **Fig. S10.** Plots for combination RND3_ENST00000263895, cg17730764 and hsa-miR-200c-3p. **Fig. S11.** Plots for combination ACOT7_ENST00000377855, cg16429975 and hsa-miR-155-5p. **Fig. S12.** Plots for combination TMTC3_ENST00000266712, cg07537152 and hsa-miR-98-5p. **Fig. S13.** Plots for combination SCD5_ENST00000319540, cg09031823 and hsa-miR-200a-3p. **Fig. S14.** Distribution of the underlying methylation probes in three regions: promoter, gene body, and 3′ UTR. **Table S1.** Combinations with significant methylation and miRNA interaction.

## Data Availability

*Transcript expression: TOIL RSEM fpkm* https://toil.xenahubs.net/download/tcga_RSEM_isoform_fpkm.gz *Clinical data: Curated clinical data.* https://pancanatlas.xenahubs.net/download/Survival_SupplementalTable_S1_20171025_xena_sp.gz *Methylation: Methylation450k.* https://tcga.xenahubs.net/download/TCGA.BLCA.sampleMap/HumanMethylation450.gz *miRNA: IlluminaHiseq.* https://tcga.xenahubs.net/download/TCGA.BLCA.sampleMap/miRNA_HiSeq_gene.gz *ID/gene mapping file:* https://tcga.xenahubs.net/download/probeMap/illuminaMethyl450_hg19_GPL16304_TCGAlegacy

## Abbreviations

TCGA: The Cancer Genome Atlas
ICGC: The International Cancer Genome Consortium
AS: Alternative splicing
KEGG: Kyoto Encyclopedia of Genes and Genomes
GDC: Genomic Data Commons
PANCAN: TCGA Pan-Cancer
BLCA: Bladder Cancer
QC: Quality control
LRT: Likelihood ratio test
LL: Low methylation, low miRNA group
HH: High methylation, high miRNA group
HL: High methylation, low miRNA group
LH: Low methylation, high miRNA group
