# Supplementary material for: Epigenetic interplay between methylation and miRNA in bladder cancer: focus on isoform expression
Source: BMC Genomics. 2021 Oct 21;22(Suppl 3):754. doi: 10.1186/s12864-021-08052-9 (PMC8529714; doi:10.1186/s12864-021-08052-9)
Supplement: Supplementary file 1 — Additional file 1 Fig. S1. Plots for combination SGCD_ENST00000435422, cg19748027 and hsa-miR-409-3p. Fig. S2. Plots for combination PLS1_ENST00000457734, cg05652551 and hsa-miR-142-5p. Fig. S3. Plots for combination CAV1_ENST00000341049, cg04474049 and hsa-miR-194-5p. Fig. S4. Plots for combination PLS1_ENST00000457734, cg05652551 and hsa-miR-155-5p. Fig. S5. Plots for combination HID1_ENST00000425042, cg07430967 and hsa-miR-125a-5p. Fig. S6. Plots for combination TGFBR3_ENST00000212355, cg08648138 and hsa-let-7c-5p. Fig. S7. Plots for combination PMEPA1_ENST00000341744, cg01515444 and hsa-miR-200a-5p. Fig. S8. Plots for combination H2AFY_ENST00000304332, cg01874869 and hsa-miR-100-5p. Fig. S9. Plots for combination THBS2_ENST00000366787, cg19681793 and hsa-miR-105-5p. Fig. S10. Plots for combination RND3_ENST00000263895, cg17730764 and hsa-miR-200c-3p. Fig. S11. Plots for combination ACOT7_ENST00000377855, cg16429975 and hsa-miR-155-5p. Fig. S12. Plots for combination TMTC3_ENST00000266712, cg07537152 and hsa-miR-98-5p. Fig. S13. Plots for combination SCD5_ENST00000319540, cg09031823 and hsa-miR-200a-3p. Fig. S14. Distribution of the underlying methylation probes in three regions: promoter, gene body, and 3′ UTR. Table S1. Combinations with significant methylation and miRNA interaction. [file 12864_2021_8052_MOESM1_ESM.docx]

**Supplementary Material**


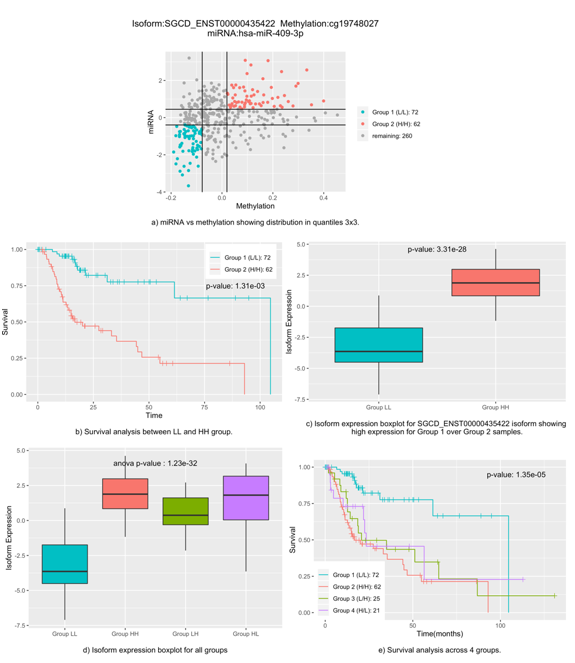


**Figure S1.** Plots for combination SGCD_ENST00000435422, cg19748027 and hsa-miR-409-3p


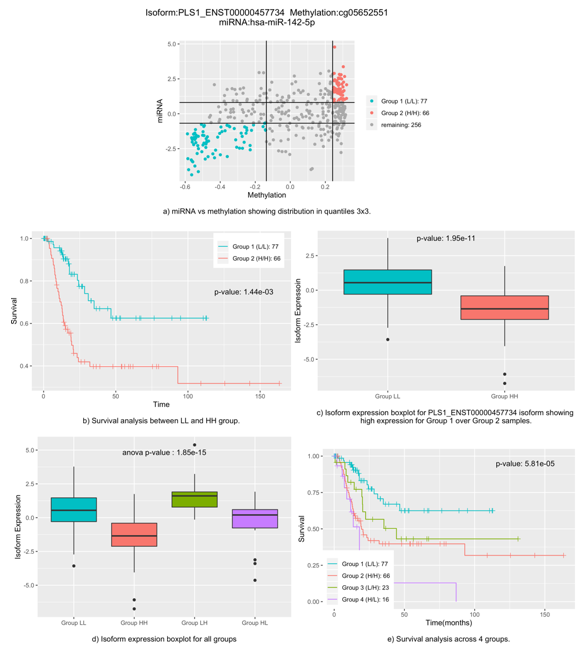


**Figure S2.** Plots for combination PLS1_ENST00000457734, cg05652551 and hsa-miR-142-5p


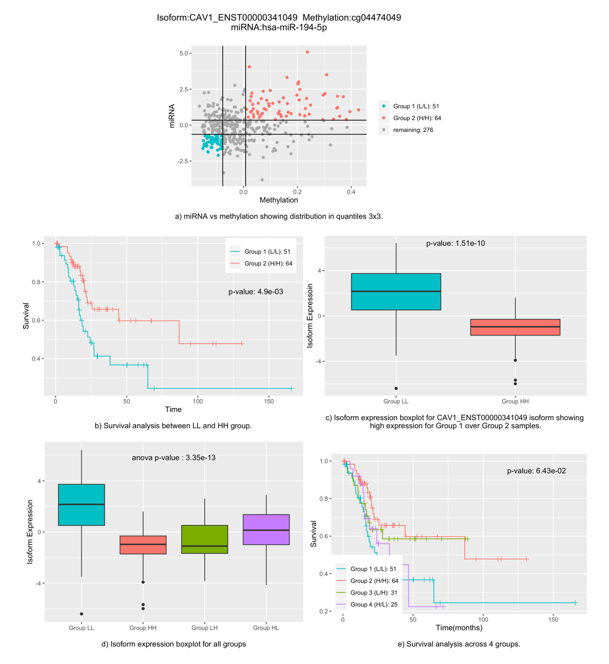


**Figure S3.** Plots for combination CAV1_ENST00000341049, cg04474049 and hsa-miR-194-5p


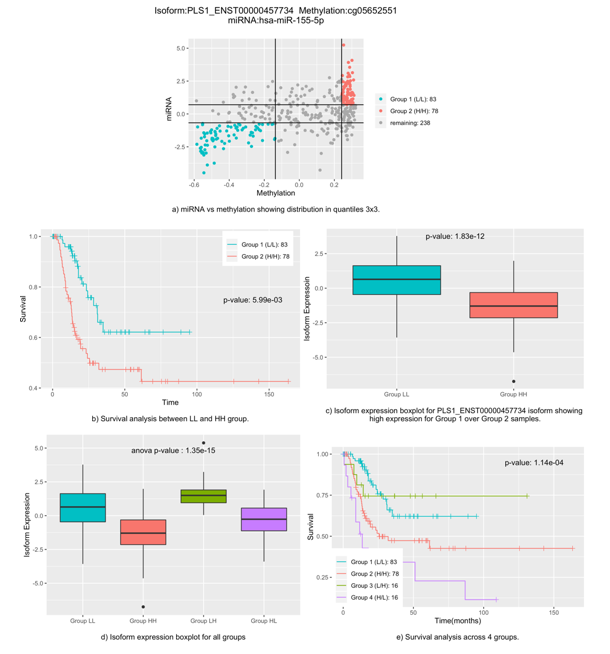


**Figure S4.** Plots for combination PLS1_ENST00000457734, cg05652551 and hsa-miR-155-5p


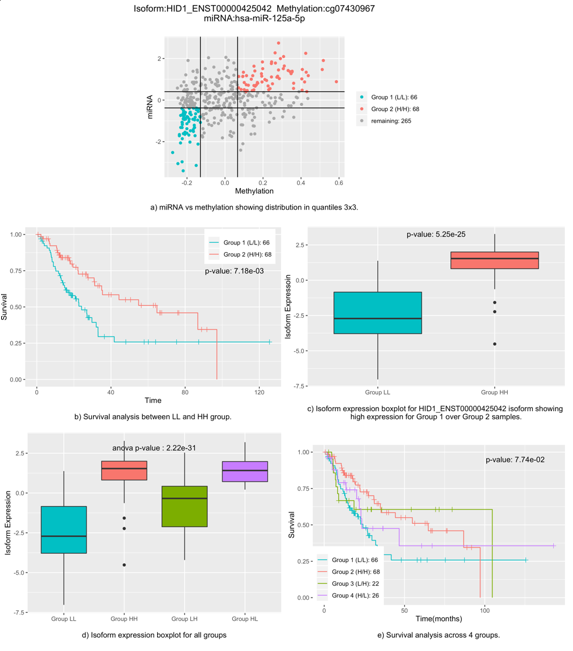


**Figure S5.** Plots for combination HID1_ENST00000425042, cg07430967 and hsa-miR-125a-5p

e
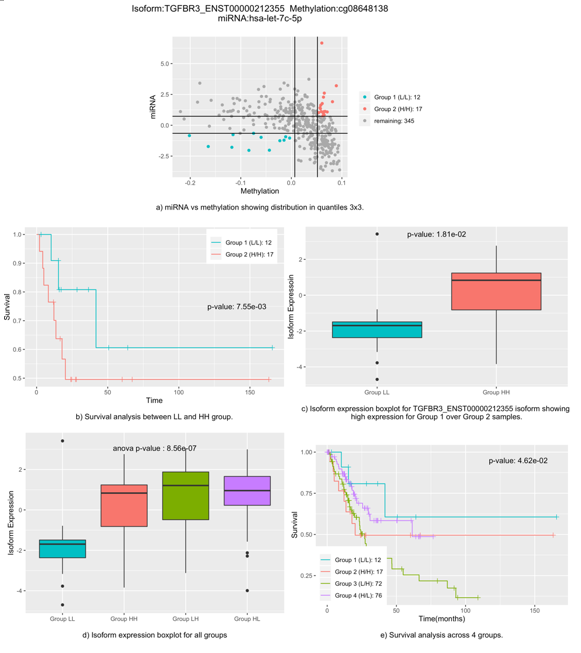


**Figure S6.** Plots for combination TGFBR3_ENST00000212355, cg08648138 and hsa-let-7c-5p


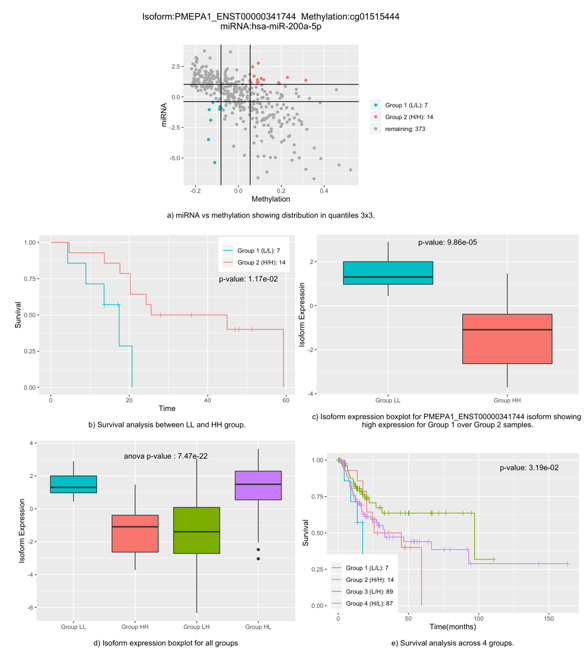


**Figure S7.** Plots for combination PMEPA1_ENST00000341744, cg01515444 and hsa-miR-200a-5p


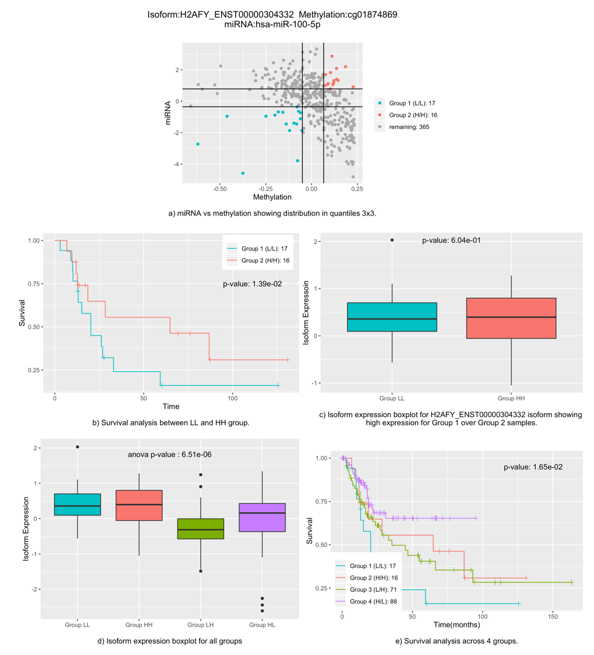


**Figure S8.** Plots for combination H2AFY_ENST00000304332, cg01874869 and hsa-miR-100-5p


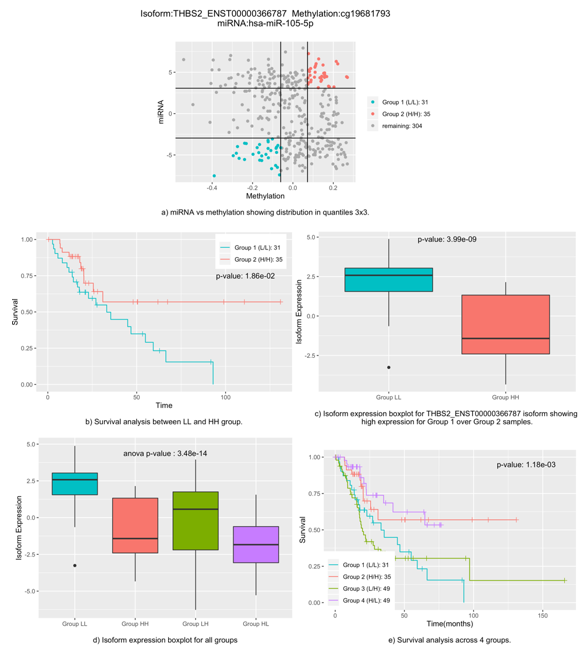


**Figure S9.** Plots for combination THBS2_ENST00000366787, cg19681793 and hsa-miR-105-5p


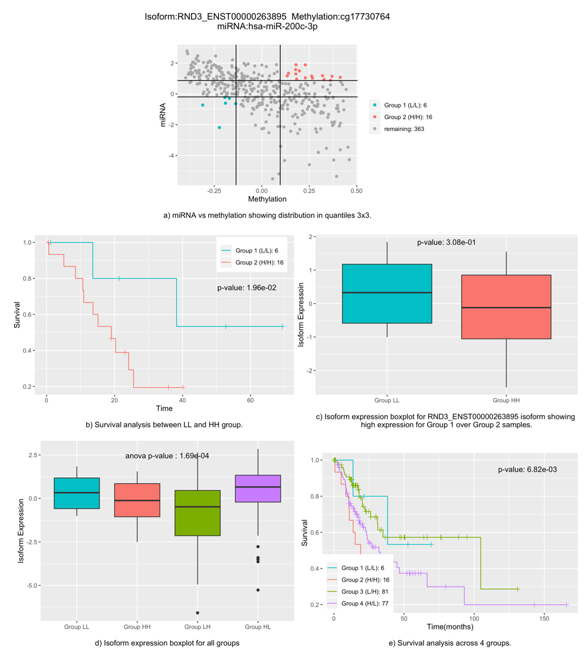


**Figure S10.** Plots for combination RND3_ENST00000263895, cg17730764 and hsa-miR-200c-3p


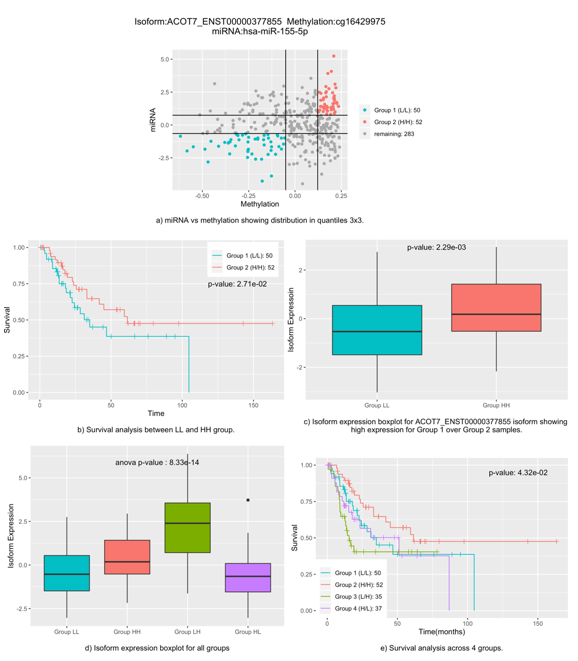


**Figure S11.** Plots for combination ACOT7_ENST00000377855, cg16429975 and hsa-miR-155-5p


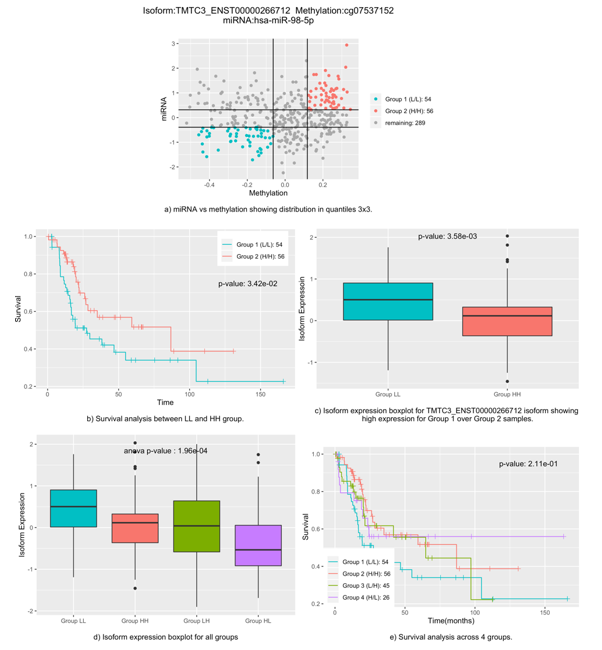


**Figure S12.** Plots for combination TMTC3_ENST00000266712, cg07537152 and hsa-miR-98-5p


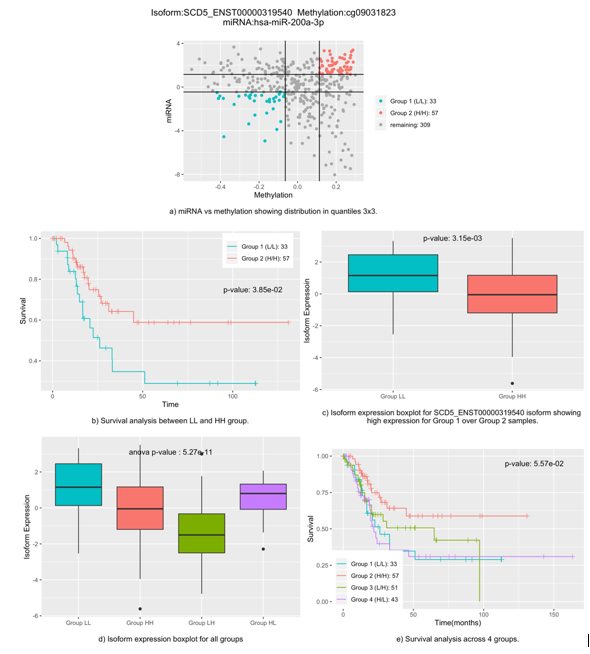


**Figure S13.** Plots for combination SCD5_ENST00000319540, cg09031823 and hsa-miR-200a-3p

**Figure S14.** Distribution of the underlying methylation probes in three regions: promoter, gene body, and 3’ UTR

**Table S1.** Combinations with significant methylation and miRNA interaction.

| Isoform | methylation | miRNA | N | pval_methylation* | pval_miRNA* | pval_lrt | bonferroni | cox_pval |
| --- | --- | --- | --- | --- | --- | --- | --- | --- |
| SGCD_ENST00000435422 | cg19748027 | hsa-miR-409-3p | 394 | 7.26E-24 | 4.17E-20 | 5.24E-10 | 1.34E-03 | 1.31E-03 |
| PLS1_ENST00000457734 | cg05652551 | hsa-miR-142-5p | 399 | 1.78E-18 | 4.43E-01 | 9.10E-10 | 2.33E-03 | 1.44E-03 |
| CAV1_ENST00000341049 | cg04474049 | hsa-miR-194-5p | 391 | 5.95E-09 | 2.02E-15 | 1.45E-08 | 3.70E-02 | 4.90E-03 |
| PLS1_ENST00000457734 | cg05652551 | hsa-miR-155-5p | 399 | 2.96E-18 | 3.68E-01 | 7.07E-12 | 1.81E-05 | 5.99E-03 |
| HID1_ENST00000425042 | cg07430967 | hsa-miR-125a-5p | 399 | 3.92E-39 | 6.58E-04 | 1.50E-12 | 3.83E-06 | 7.18E-03 |
| TGFBR3_ENST00000212355 | cg08648138 | hsa-let-7c-5p | 374 | 7.82E-08 | 1.03E-06 | 9.24E-13 | 2.37E-06 | 7.55E-03 |
| PMEPA1_ENST00000341744 | cg01515444 | hsa-miR-200a-5p | 394 | 9.50E-02 | 2.26E-19 | 2.20E-10 | 5.63E-04 | 1.17E-02 |
| H2AFY_ENST00000304332 | cg01874869 | hsa-miR-100-5p | 398 | 9.22E-01 | 5.94E-04 | 1.24E-08 | 3.18E-02 | 1.39E-02 |
| THBS2_ENST00000366787 | cg19681793 | hsa-miR-105-5p | 370 | 1.68E-26 | 5.74E-03 | 1.29E-09 | 3.31E-03 | 1.86E-02 |
| RND3_ENST00000263895 | cg17730764 | hsa-miR-200c-3p | 385 | 4.00E-01 | 1.01E-04 | 9.19E-09 | 2.35E-02 | 1.96E-02 |
| ACOT7_ENST00000377855 | cg16429975 | hsa-miR-155-5p | 385 | 1.62E-11 | 2.12E-19 | 1.82E-08 | 4.66E-02 | 2.71E-02 |
| TMTC3_ENST00000266712 | cg07537152 | hsa-miR-98-5p | 399 | 2.44E-11 | 2.75E-02 | 6.13E-09 | 1.57E-02 | 3.42E-02 |
| SCD5_ENST00000319540 | cg09031823 | hsa-miR-200a-3p | 399 | 2.44E-01 | 6.19E-17 | 2.56E-11 | 6.56E-05 | 3.85E-02 |
| ELOVL6_ENST00000394607 | cg11320318 | hsa-miR-142-5p | 399 | 1.77E-25 | 1.72E-01 | 1.36E-09 | 3.48E-03 | 5.32E-02 |
| DHCR24_ENST00000371269 | cg15961901 | hsa-miR-191-5p | 396 | 7.19E-01 | 2.28E-06 | 6.41E-10 | 1.64E-03 | 6.79E-02 |
| ELOVL6_ENST00000394607 | cg09934926 | hsa-miR-142-5p | 399 | 6.48E-26 | 6.77E-02 | 5.21E-09 | 1.33E-02 | 7.14E-02 |
| PMEPA1_ENST00000341744 | cg22469870 | hsa-miR-200a-5p | 394 | 5.46E-01 | 1.04E-26 | 1.08E-12 | 2.78E-06 | 7.21E-02 |
| TRAK1_ENST00000341421 | cg20372886 | hsa-miR-324-3p | 398 | 6.58E-12 | 2.45E-04 | 4.70E-09 | 1.20E-02 | 8.11E-02 |
| PMEPA1_ENST00000395816 | cg20208990 | hsa-miR-200b-5p | 368 | 5.07E-01 | 2.45E-17 | 3.69E-10 | 9.45E-04 | 8.12E-02 |
| PMEPA1_ENST00000341744 | cg20208990 | hsa-miR-200b-5p | 390 | 6.36E-01 | 1.63E-26 | 2.39E-10 | 6.11E-04 | 1.13E-01 |
| TIMP2_ENST00000585421 | cg01502428 | hsa-miR-200c-3p | 388 | 6.40E-15 | 3.59E-20 | 8.19E-12 | 2.10E-05 | 1.32E-01 |
| PMEPA1_ENST00000395816 | cg22469870 | hsa-miR-200a-5p | 372 | 2.79E-01 | 5.05E-17 | 1.31E-09 | 3.36E-03 | 1.35E-01 |
| SHROOM3_ENST00000296043 | cg09640555 | hsa-miR-766-3p | 399 | 3.68E-02 | 1.73E-01 | 5.48E-09 | 1.40E-02 | 1.35E-01 |
| SLC15A2_ENST00000489711 | cg02034887 | hsa-miR-378a-5p | 399 | 2.59E-08 | 3.15E-03 | 9.00E-10 | 2.31E-03 | 1.37E-01 |
| SGCD_ENST00000435422 | cg25232043 | hsa-miR-511-5p | 397 | 9.42E-19 | 5.72E-01 | 3.28E-09 | 8.41E-03 | 1.46E-01 |
| TIMP2_ENST00000585421 | cg04689178 | hsa-miR-200c-3p | 388 | 9.61E-02 | 1.07E-37 | 5.94E-13 | 1.52E-06 | 1.51E-01 |
| TNRC18_ENST00000399537 | cg04348816 | hsa-miR-1307-3p | 393 | 7.52E-06 | 3.24E-01 | 1.81E-08 | 4.65E-02 | 1.65E-01 |
| PMEPA1_ENST00000341744 | cg26912636 | hsa-miR-200a-5p | 394 | 1.65E-02 | 4.38E-30 | 7.50E-10 | 1.92E-03 | 1.69E-01 |
| SVIL_ENST00000375398 | cg26508239 | hsa-miR-18a-3p | 389 | 1.42E-04 | 6.84E-10 | 8.57E-11 | 2.19E-04 | 1.83E-01 |
| NFIC_ENST00000589123 | cg03605666 | hsa-miR-15b-5p | 399 | 4.38E-02 | 6.49E-01 | 5.37E-12 | 1.38E-05 | 1.86E-01 |
| SYT7_ENST00000263846 | cg23370512 | hsa-miR-483-5p | 367 | 5.35E-04 | 7.16E-03 | 1.55E-09 | 3.97E-03 | 1.87E-01 |
| TACC1_ENST00000276520 | cg04889181 | hsa-miR-193b-3p | 399 | 2.91E-02 | 2.74E-04 | 3.17E-09 | 8.12E-03 | 2.12E-01 |
| PMEPA1_ENST00000347215 | cg20208990 | hsa-miR-200b-5p | 341 | 9.86E-03 | 4.95E-13 | 1.92E-08 | 4.91E-02 | 2.23E-01 |
| PMEPA1_ENST00000395816 | cg08567517 | hsa-miR-200b-5p | 368 | 8.55E-01 | 2.77E-17 | 7.09E-09 | 1.82E-02 | 2.27E-01 |
| PMEPA1_ENST00000341744 | cg05114898 | hsa-miR-200a-5p | 394 | 1.95E-03 | 1.09E-18 | 6.04E-14 | 1.55E-07 | 2.28E-01 |
| OSBPL10_ENST00000396556 | cg23429042 | hsa-miR-155-5p | 398 | 6.75E-05 | 3.54E-01 | 4.76E-10 | 1.22E-03 | 2.32E-01 |
| FANCI_ENST00000310775 | cg03489186 | hsa-let-7c-5p | 396 | 5.65E-01 | 5.68E-01 | 2.83E-09 | 7.25E-03 | 2.34E-01 |
| FN1_ENST00000456923 | cg09166091 | hsa-miR-200c-3p | 383 | 4.11E-01 | 1.61E-35 | 9.24E-10 | 2.37E-03 | 2.57E-01 |
| DHCR24_ENST00000371269 | cg16051114 | hsa-miR-191-5p | 396 | 2.11E-01 | 3.24E-06 | 1.31E-09 | 3.35E-03 | 2.77E-01 |
| ACTA2_ENST00000224784 | cg09990481 | hsa-miR-484 | 396 | 7.70E-30 | 4.47E-14 | 1.08E-08 | 2.78E-02 | 2.79E-01 |
| MYLK_ENST00000578202 | cg07638500 | hsa-miR-146b-5p | 387 | 1.22E-06 | 6.10E-01 | 1.24E-09 | 3.19E-03 | 3.11E-01 |
| PMEPA1_ENST00000341744 | cg00177431 | hsa-miR-200a-5p | 394 | 3.94E-01 | 3.56E-31 | 1.33E-14 | 3.40E-08 | 3.12E-01 |
| SCD5_ENST00000319540 | cg08030987 | hsa-miR-141-3p | 393 | 5.66E-07 | 6.37E-08 | 1.15E-08 | 2.94E-02 | 3.13E-01 |
| FN1_ENST00000456923 | cg24723421 | hsa-miR-200b-3p | 365 | 1.96E-02 | 7.03E-30 | 5.90E-09 | 1.51E-02 | 3.20E-01 |
| FBN1_ENST00000559133 | cg25505106 | hsa-miR-29c-3p | 399 | 2.65E-11 | 9.90E-10 | 1.45E-08 | 3.70E-02 | 3.31E-01 |
| CARD11_ENST00000396946 | cg23352157 | hsa-miR-146b-5p | 398 | 2.47E-27 | 1.27E-10 | 8.06E-13 | 2.06E-06 | 3.59E-01 |
| CNN3_ENST00000370206 | cg22608160 | hsa-miR-142-3p | 386 | 7.15E-03 | 2.45E-01 | 9.76E-09 | 2.50E-02 | 3.68E-01 |
| HOPX_ENST00000503639 | cg06771126 | hsa-miR-1228-3p | 295 | 2.07E-11 | 6.48E-07 | 5.83E-09 | 1.49E-02 | 3.69E-01 |
| CXCL12_ENST00000374429 | cg03916630 | hsa-miR-362-3p | 394 | 3.42E-02 | 2.31E-01 | 7.17E-09 | 1.84E-02 | 3.79E-01 |
| PMEPA1_ENST00000341744 | cg08567517 | hsa-miR-200a-5p | 394 | 2.36E-01 | 1.53E-29 | 9.78E-10 | 2.50E-03 | 3.87E-01 |
| CALU_ENST00000249364 | cg13169491 | hsa-miR-155-5p | 398 | 4.84E-03 | 6.79E-07 | 9.34E-10 | 2.39E-03 | 3.89E-01 |
| PMEPA1_ENST00000341744 | cg26467279 | hsa-miR-200a-5p | 394 | 3.38E-04 | 3.54E-19 | 3.19E-11 | 8.18E-05 | 3.94E-01 |
| BCAT1_ENST00000261192 | cg16490209 | hsa-miR-155-5p | 399 | 1.41E-06 | 4.38E-12 | 1.92E-11 | 4.93E-05 | 4.01E-01 |
| NFIC_ENST00000589123 | cg04251208 | hsa-miR-15b-5p | 398 | 7.94E-01 | 9.07E-03 | 9.36E-13 | 2.40E-06 | 4.11E-01 |
| CLIC4_ENST00000374379 | cg15649857 | hsa-miR-142-3p | 399 | 6.09E-24 | 8.98E-01 | 4.29E-13 | 1.10E-06 | 4.20E-01 |
| SHROOM3_ENST00000296043 | cg09640555 | hsa-miR-338-3p | 398 | 7.47E-03 | 3.02E-02 | 1.88E-08 | 4.82E-02 | 4.25E-01 |
| CLIC4_ENST00000374379 | cg15649857 | hsa-miR-155-5p | 399 | 5.90E-19 | 1.35E-01 | 1.75E-13 | 4.49E-07 | 4.29E-01 |
| PXDC1_ENST00000380277 | cg03758047 | hsa-miR-939-5p | 373 | 5.20E-04 | 8.76E-05 | 7.89E-09 | 2.02E-02 | 4.36E-01 |
| IFITM1_ENST00000408968 | cg03038262 | hsa-miR-130a-3p | 399 | 2.86E-23 | 1.54E-05 | 4.05E-09 | 1.04E-02 | 4.41E-01 |
| XPR1_ENST00000367590 | cg15392819 | hsa-miR-150-5p | 399 | 8.18E-05 | 1.43E-02 | 1.16E-08 | 2.98E-02 | 4.45E-01 |
| SCD5_ENST00000319540 | cg05672616 | hsa-miR-200a-3p | 399 | 4.33E-07 | 3.05E-15 | 2.39E-11 | 6.12E-05 | 4.52E-01 |
| PMEPA1_ENST00000395816 | cg20208990 | hsa-miR-200a-5p | 372 | 9.08E-01 | 1.26E-17 | 1.09E-10 | 2.80E-04 | 4.55E-01 |
| TGFBR3_ENST00000212355 | cg08648138 | hsa-miR-15b-5p | 374 | 6.84E-10 | 1.22E-06 | 5.23E-09 | 1.34E-02 | 4.57E-01 |
| CD109_ENST00000287097 | cg23004174 | hsa-miR-155-5p | 399 | 3.30E-08 | 9.98E-19 | 1.47E-08 | 3.78E-02 | 4.57E-01 |
| CD109_ENST00000422508 | cg23004174 | hsa-miR-155-5p | 399 | 1.02E-01 | 5.84E-09 | 1.50E-08 | 3.84E-02 | 4.57E-01 |
| SCD5_ENST00000319540 | cg18948646 | hsa-miR-200a-3p | 399 | 8.41E-09 | 8.16E-13 | 2.50E-11 | 6.40E-05 | 4.67E-01 |
| SCD5_ENST00000319540 | cg04237666 | hsa-miR-200a-3p | 399 | 1.45E-05 | 2.69E-14 | 4.81E-11 | 1.23E-04 | 4.73E-01 |
| PMEPA1_ENST00000347215 | cg20208990 | hsa-miR-200a-5p | 345 | 1.21E-02 | 8.28E-15 | 9.81E-09 | 2.51E-02 | 4.73E-01 |
| PMEPA1_ENST00000341744 | cg10100887 | hsa-miR-200a-5p | 394 | 1.20E-01 | 4.12E-22 | 2.73E-11 | 6.99E-05 | 4.85E-01 |
| RAVER2_ENST00000371072 | cg10378032 | hsa-miR-615-3p | 294 | 1.41E-15 | 3.91E-06 | 3.85E-09 | 9.87E-03 | 4.86E-01 |
| TIMP2_ENST00000585421 | cg15010903 | hsa-miR-200c-3p | 388 | 1.63E-23 | 6.35E-17 | 3.35E-10 | 8.59E-04 | 4.88E-01 |
| MXRA7_ENST00000449428 | cg18755226 | hsa-miR-940 | 389 | 6.58E-01 | 4.16E-14 | 6.88E-09 | 1.76E-02 | 4.92E-01 |
| PMEPA1_ENST00000341744 | cg00626515 | hsa-miR-200a-5p | 394 | 1.56E-02 | 4.26E-23 | 1.21E-10 | 3.11E-04 | 5.07E-01 |
| ZCCHC24_ENST00000372336 | cg05691833 | hsa-miR-532-3p | 390 | 5.66E-10 | 2.39E-12 | 2.20E-09 | 5.64E-03 | 5.47E-01 |
| OSBPL10_ENST00000396556 | cg16165903 | hsa-miR-155-5p | 399 | 7.48E-04 | 6.30E-01 | 6.75E-09 | 1.73E-02 | 5.59E-01 |
| PMEPA1_ENST00000341744 | cg22469870 | hsa-miR-200b-5p | 390 | 1.60E-01 | 4.00E-21 | 6.52E-09 | 1.67E-02 | 5.62E-01 |
| SEMA3E_ENST00000307792 | cg19542238 | hsa-miR-17-3p | 384 | 6.83E-02 | 4.60E-12 | 3.57E-09 | 9.15E-03 | 5.70E-01 |
| PMEPA1_ENST00000341744 | cg20208990 | hsa-miR-200a-5p | 394 | 2.67E-01 | 1.21E-30 | 3.06E-12 | 7.84E-06 | 5.71E-01 |
| TIMP2_ENST00000585421 | cg25344194 | hsa-miR-200c-3p | 388 | 4.61E-07 | 1.81E-27 | 8.86E-09 | 2.27E-02 | 5.86E-01 |
| ATP11A_ENST00000375630 | cg09507215 | hsa-miR-769-5p | 394 | 1.17E-01 | 9.83E-02 | 4.18E-09 | 1.07E-02 | 5.94E-01 |
| IRF4_ENST00000380956 | cg11307823 | hsa-miR-125b-5p | 399 | 1.70E-24 | 8.99E-01 | 3.48E-10 | 8.92E-04 | 6.09E-01 |
| PMEPA1_ENST00000395816 | cg17978103 | hsa-miR-200b-5p | 368 | 2.49E-01 | 2.19E-17 | 1.36E-08 | 3.50E-02 | 6.10E-01 |
| SGCD_ENST00000435422 | cg11404992 | hsa-miR-511-5p | 398 | 2.56E-35 | 5.04E-02 | 4.41E-11 | 1.13E-04 | 6.14E-01 |
| ADCY9_ENST00000294016 | cg06153873 | hsa-miR-185-3p | 363 | 8.46E-03 | 3.32E-11 | 1.76E-08 | 4.52E-02 | 6.14E-01 |
| LY6K_ENST00000292430 | cg19413366 | hsa-miR-155-5p | 389 | 9.70E-36 | 7.57E-09 | 7.50E-09 | 1.92E-02 | 6.21E-01 |
| TIMP2_ENST00000585421 | cg04999637 | hsa-miR-200c-3p | 386 | 1.23E-01 | 1.38E-38 | 5.53E-09 | 1.42E-02 | 6.32E-01 |
| PMEPA1_ENST00000341744 | cg00138126 | hsa-miR-200a-5p | 394 | 7.57E-02 | 3.30E-25 | 4.58E-09 | 1.17E-02 | 6.36E-01 |
| PMEPA1_ENST00000341744 | cg07143805 | hsa-miR-200a-5p | 394 | 3.73E-02 | 4.81E-24 | 2.56E-09 | 6.55E-03 | 6.38E-01 |
| PMEPA1_ENST00000341744 | cg17978103 | hsa-miR-200b-5p | 390 | 6.22E-01 | 2.93E-25 | 2.34E-09 | 5.99E-03 | 6.40E-01 |
| OSBPL10_ENST00000396556 | cg27590407 | hsa-miR-155-5p | 399 | 6.84E-05 | 7.61E-01 | 3.05E-09 | 7.80E-03 | 6.47E-01 |
| PMEPA1_ENST00000341744 | cg10100887 | hsa-miR-200b-5p | 390 | 1.65E-02 | 2.11E-18 | 5.55E-09 | 1.42E-02 | 6.60E-01 |
| PMEPA1_ENST00000341744 | cg00177431 | hsa-miR-200b-5p | 390 | 4.63E-01 | 8.84E-26 | 1.46E-11 | 3.75E-05 | 6.63E-01 |
| ATP11A_ENST00000375630 | cg18475128 | hsa-miR-769-5p | 394 | 2.09E-03 | 2.47E-01 | 6.98E-09 | 1.79E-02 | 6.76E-01 |
| PMEPA1_ENST00000341744 | cg08243465 | hsa-miR-200b-5p | 389 | 6.98E-02 | 4.43E-23 | 5.94E-10 | 1.52E-03 | 6.85E-01 |
| THRA_ENST00000450525 | cg09304381 | hsa-miR-185-3p | 399 | 1.84E-01 | 1.59E-01 | 9.68E-09 | 2.48E-02 | 6.86E-01 |
| CLIC4_ENST00000374379 | cg07201717 | hsa-miR-155-5p | 397 | 6.88E-05 | 1.17E-07 | 2.21E-10 | 5.65E-04 | 6.91E-01 |
| MYLK_ENST00000360772 | cg26394191 | hsa-miR-589-5p | 399 | 5.50E-18 | 4.86E-12 | 6.48E-09 | 1.66E-02 | 7.15E-01 |
| PMEPA1_ENST00000341744 | cg25840824 | hsa-miR-200b-5p | 390 | 4.18E-05 | 4.38E-30 | 5.50E-09 | 1.41E-02 | 7.38E-01 |
| MYLK_ENST00000360772 | cg23184556 | hsa-miR-181a-2-3p | 399 | 1.87E-10 | 1.15E-05 | 9.98E-12 | 2.56E-05 | 7.58E-01 |
| MYLK_ENST00000418370 | cg23184556 | hsa-miR-181a-2-3p | 399 | 9.96E-05 | 6.05E-04 | 1.93E-08 | 4.94E-02 | 7.58E-01 |
| PMEPA1_ENST00000341744 | cg16178855 | hsa-miR-200a-5p | 394 | 8.37E-05 | 2.35E-34 | 1.97E-10 | 5.05E-04 | 7.70E-01 |
| SCD5_ENST00000319540 | cg09363604 | hsa-miR-200a-3p | 399 | 8.12E-11 | 5.37E-09 | 8.75E-10 | 2.24E-03 | 7.78E-01 |
| ERBB2_ENST00000541774 | cg06185555 | hsa-miR-205-5p | 393 | 9.58E-33 | 2.86E-01 | 1.12E-08 | 2.86E-02 | 7.99E-01 |
| TPD52L1_ENST00000392482 | cg05572930 | hsa-miR-200b-3p | 391 | 8.94E-01 | 1.38E-10 | 1.88E-08 | 4.81E-02 | 8.03E-01 |
| PMEPA1_ENST00000341744 | cg25840824 | hsa-miR-200a-5p | 394 | 1.67E-07 | 4.22E-36 | 2.00E-11 | 5.13E-05 | 8.03E-01 |
| PMEPA1_ENST00000341744 | cg01117384 | hsa-miR-200b-5p | 390 | 4.92E-01 | 2.86E-25 | 5.60E-09 | 1.43E-02 | 8.05E-01 |
| TNS1_ENST00000171887 | cg05386769 | hsa-miR-130b-5p | 399 | 1.43E-10 | 1.33E-26 | 4.74E-10 | 1.21E-03 | 8.11E-01 |
| TGFBR3_ENST00000212355 | cg10324998 | hsa-miR-15b-5p | 388 | 7.93E-11 | 2.43E-03 | 4.25E-09 | 1.09E-02 | 8.23E-01 |
| TGFBR3_ENST00000212355 | cg10324998 | hsa-miR-16-5p | 388 | 5.99E-10 | 1.38E-05 | 5.76E-09 | 1.48E-02 | 8.31E-01 |
| CLIC4_ENST00000374379 | cg07201717 | hsa-miR-142-3p | 397 | 4.94E-07 | 1.07E-04 | 4.18E-09 | 1.07E-02 | 8.31E-01 |
| SMAD6_ENST00000288840 | cg17232357 | hsa-miR-134-5p | 398 | 8.12E-02 | 6.75E-04 | 3.79E-10 | 9.70E-04 | 8.32E-01 |
| PMEPA1_ENST00000395816 | cg12514933 | hsa-miR-200b-5p | 368 | 3.19E-03 | 2.97E-13 | 8.50E-09 | 2.18E-02 | 8.37E-01 |
| NFIC_ENST00000589123 | cg20714551 | hsa-miR-15b-5p | 399 | 4.43E-08 | 7.14E-01 | 8.74E-09 | 2.24E-02 | 8.43E-01 |
| LY6K_ENST00000292430 | cg00497110 | hsa-miR-155-5p | 391 | 2.12E-43 | 6.16E-08 | 3.39E-09 | 8.69E-03 | 8.53E-01 |
| ZCCHC24_ENST00000372336 | cg13834460 | hsa-miR-532-3p | 392 | 2.49E-13 | 1.41E-07 | 1.39E-10 | 3.56E-04 | 8.55E-01 |
| ADCY9_ENST00000294016 | cg02520816 | hsa-miR-181b-5p | 352 | 3.50E-02 | 6.20E-09 | 1.74E-09 | 4.45E-03 | 8.58E-01 |
| ACOX1_ENST00000293217 | cg26205890 | hsa-miR-155-5p | 399 | 5.65E-30 | 8.54E-01 | 1.08E-09 | 2.76E-03 | 8.76E-01 |
| TIMP2_ENST00000585421 | cg20761853 | hsa-miR-200c-3p | 388 | 6.40E-20 | 8.97E-15 | 3.38E-09 | 8.64E-03 | 8.93E-01 |
| PMEPA1_ENST00000395816 | cg00177431 | hsa-miR-200a-5p | 372 | 9.24E-01 | 8.71E-17 | 2.71E-09 | 6.95E-03 | 9.06E-01 |
| ZNF175_ENST00000262259 | cg10668363 | hsa-miR-374b-5p | 399 | 2.84E-01 | 6.82E-02 | 1.92E-09 | 4.91E-03 | 9.12E-01 |
| ACOX1_ENST00000293217 | cg16024937 | hsa-miR-155-5p | 399 | 6.39E-32 | 1.28E-01 | 2.00E-12 | 5.13E-06 | 9.22E-01 |
| RAB23_ENST00000317483 | cg03794840 | hsa-miR-15b-5p | 398 | 2.51E-16 | 5.84E-09 | 4.28E-09 | 1.10E-02 | 9.33E-01 |
| TGFBR3_ENST00000212355 | cg08648138 | hsa-miR-15a-5p | 374 | 6.33E-08 | 1.03E-05 | 6.06E-09 | 1.55E-02 | 9.46E-01 |
| PMEPA1_ENST00000347215 | cg26467279 | hsa-miR-200a-5p | 345 | 3.01E-02 | 3.09E-10 | 6.19E-09 | 1.58E-02 | 9.55E-01 |
| SVIL_ENST00000375398 | cg24726783 | hsa-miR-18a-3p | 379 | 1.91E-07 | 2.48E-08 | 5.23E-09 | 1.34E-02 | 9.58E-01 |
| ACOX1_ENST00000293217 | cg26205890 | hsa-miR-150-5p | 399 | 2.31E-45 | 7.20E-05 | 1.85E-08 | 4.73E-02 | 9.60E-01 |
| PMEPA1_ENST00000341744 | cg08243465 | hsa-miR-200a-5p | 393 | 3.21E-01 | 1.43E-25 | 5.37E-10 | 1.38E-03 | 9.68E-01 |
| LY6K_ENST00000292430 | cg15164446 | hsa-miR-155-5p | 391 | 2.49E-44 | 2.07E-10 | 6.10E-09 | 1.56E-02 | 9.71E-01 |
| SCD5_ENST00000319540 | cg08030987 | hsa-miR-200a-3p | 399 | 1.77E-05 | 6.71E-12 | 6.55E-12 | 1.68E-05 | 9.71E-01 |
| MXRA7_ENST00000449428 | cg00004089 | hsa-miR-940 | 396 | 1.46E-01 | 1.19E-14 | 3.91E-09 | 1.00E-02 | 9.72E-01 |
| FN1_ENST00000456923 | cg21494132 | hsa-miR-200c-3p | 388 | 1.88E-04 | 2.92E-27 | 1.00E-09 | 2.56E-03 | 9.81E-01 |
| PMEPA1_ENST00000341744 | cg05114898 | hsa-miR-200b-5p | 390 | 2.83E-06 | 7.94E-15 | 4.79E-11 | 1.23E-04 | 9.88E-01 |
| FN1_ENST00000456923 | cg21494132 | hsa-miR-200b-3p | 397 | 1.05E-04 | 6.98E-30 | 8.36E-12 | 2.14E-05 | 9.89E-01 |
| PMEPA1_ENST00000347215 | cg00626515 | hsa-miR-200a-5p | 345 | 1.98E-01 | 1.05E-11 | 1.02E-08 | 2.62E-02 | 9.92E-01 |
| PMEPA1_ENST00000341744 | cg17978103 | hsa-miR-200a-5p | 394 | 1.46E-01 | 7.30E-30 | 8.79E-12 | 2.25E-05 | 9.98E-01 |
| PMEPA1_ENST00000395816 | cg00138126 | hsa-miR-200a-5p | 372 | 1.90E-02 | 5.98E-14 | 8.33E-09 | 2.13E-02 | 9.99E-01 |

* The p-values for terms in full model
